# Low-Frequency Magnetic Sensing Using Magnetically Modulated Microcavity Resonant Mode

**DOI:** 10.3390/mi16040405

**Published:** 2025-03-29

**Authors:** Xinrong Yang, Jiamin Rong, Enbo Xing, Jianglong Li, Yujie Zhang, Yanru Zhou, Wenyao Liu, Huanfei Wen, Jun Tang, Jun Liu

**Affiliations:** 1 Key Laboratory of Dynamic Testing Technology, School of Instrument and Electronics, North University of China, Taiyuan 030051, China; 15835227319@163.com (X.Y.); xiaoxing1228@126.com (E.X.); 15735657168@163.com (J.L.); zhouyanru@nuc.edu.cn (Y.Z.); liuwenyao@nuc.edu.cn (W.L.); wenhuanfei@nuc.edu.cn (H.W.); liuj@nuc.edu.cn (J.L.); 2 Key Laboratory of Widegap Semiconductor Optoelectronic Materials and Technologies, School of Semiconductors and Physics, North University of China, Taiyuan 030051, China; zayujie@126.com (Y.Z.); tangjun@nuc.edu.cn (J.T.)

**Keywords:** whispering gallery mode, microcavity, low-frequency magnetic sensing, resonant mode

## Abstract

We propose a low-frequency magnetic sensing method using a magnetically modulated microcavity resonant mode. Our magnetically sensitive unit with periodically changing magnetic poles is formed by combining an AC excitation coil with a microcavity. The microcavity vibrates at the frequency of the AC amplitude-modulated signal and changes its resonant mode when the sensing unit interacts with a low-frequency magnetic field. Signal processing is performed on the resonant spectrum to obtain low-frequency magnetic signals. The results of the experiment show that the measured sensitivity to a 0.5 Hz magnetic field is 12.49 V/mT, and a bias instability noise of 16.71 nT is achieved. We have extended the measurable frequency range of the whispering gallery mode microcavity magnetometer and presented a development in microcavity magnetic sensing and optical readout.

## 1. Introduction

Whispering gallery mode (WGM) microcavities benefit from the strong coherence of laser energy and thus exhibit high internal photon energy density. Due to the strong interaction between light and matter, small changes in the shape of the WGM microcavity are accompanied by drastic changes in the light propagation characteristics and the intensity of the optical field, making it one of the most ideal high-precision sensing platforms [1,2,3,4,5,6,7,8,9]. In the last decade, high-sensitivity applications of various physical properties, including acoustic pressure [10], acceleration [11,12], temperature [13,14], and electromagnetic field [15], have been carried out using WGM microcavities based on a high quality (Q) factor. Among them, the optomechanical magnetometer with a WGM microcavity as a carrier applies vibration and mechanical energy to the cavity through the transmission of field force by magnetostriction or a magnetic device [16,17,18]. Applications of microcavities in combination with magnetic devices such as neodymium–iron–boron permanent magnets [19], magnetostrictive materials (Terfenol-D, YIG et al.) [20,21,22], and metal oxide materials [23] have been demonstrated in other studies. The sensing mechanism is mostly based on the dispersion coupling response [24], which utilizes the high sensitivity of the mode to propagation path changes during total internal reflection and constructive interference of the laser in the microcavity and provides information on the external magnetic field by detecting the resonant frequency drift and changes in WGM and power spectral density (PSD) due to the change in cavity length. These studies have achieved extremely high sensitivity in the high-frequency and -bandwidth range.

However, this type of magnetic sensing has rarely been reported at low frequencies, mainly due to the fact that the magnetic devices mentioned above can only vibrate passively following the frequencies of external magnetic fields and often only achieve the strongest response when testing magnetic fields that match the microcavity mechanical resonance frequencies. In contrast, low-frequency magnetic fields exhibit a significant decrease in response due to their distance from the mechanical resonant frequencies. In addition, from the perspective of noise, on the one hand, the low-frequency thermal noise caused by temperature changes is difficult to eliminate because high-Q-factor microcavities highly easily undergo thermal accumulation during the process of photonic localization from total internal reflection [25]. On the other hand, the 1/*f* flicker noise intensity caused by electrical factors increases with decreasing frequency [26], which has a nonnegligible effect on magnetic signal sensing in the hertz to millihertz range. The noise generated during low-frequency magnetic sensing may drown out the test signal, limiting the development of microcavity magnetometers for low-frequency detection.

In this work, we propose a low-frequency magnetic sensing method using a magnetically modulated WGM microcavity resonant mode. By tightly bonding the AC excitation coil with the microcavity and applying a sinusoidal signal of the frequency corresponding to the strongest mechanical resonant response of the microcavity, a small-scale AC magnetic field is formed near the microcavity. When a low-frequency magnetic field to be tested is applied, the two magnetic field signals are converted into a high-frequency amplitude-modulated (AM) magnetic field force signal. The microcavity vibrates at the frequency of the AM signal, causing mechanical deformation and changes in the resonant mode. The results of the experiment show that the resonant transmission spectrum restores the test signal and exhibits a certain degree of noise suppression after demodulation and filtering. Our WGM magnetometer has a sensitivity of 12.49 V/mT for 0.5 Hz magnetic field sensing and a bias instability noise of 16.71 nT, achieving high-precision measurement of low-frequency AC magnetic fields. This method of enabling low-frequency magnetic sensing through the indirect action of magnetism with modulation and demodulation techniques reveals new prospects for WGM magnetometers.

## 2. Theory and Simulation

**Figure 1:**
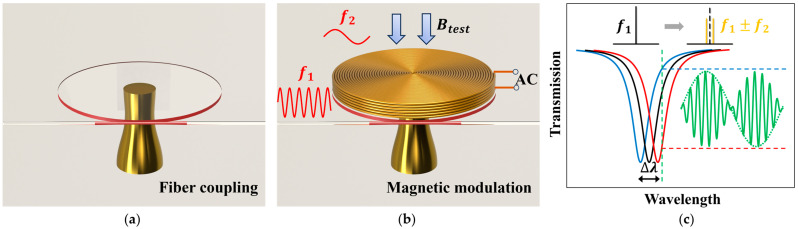
(**a**) Schematic image of the WGM microcavity with fiber coupling; the red color on the cylindrical surface of the microcavity represents the total internal reflection of the laser. (**b**) Schematic image of the modulation effect of the magnetic field generated by the excitation coil on the magnetic field to be tested. (**c**) WGM spectral shift and the generation of AM signals.

Calcium fluoride (CaF_2_) became the preferred choice for our microcavity’s material due to its low refractive index and ease of coupling. The microcavity (Figure 1a) prepared from CaF_2_ has very high optical Q factor, and higher Q indicates stronger energy storage capacity and lower light propagation loss, so the high-Q-factor microcavity can exhibit significantly improved sensitivity. CaF_2_ crystalline microcavities can be directly applied to the measurement of properties such as temperature and sound pressure through thermal expansion and simple mechanical resonance. However, due to their electromagnetic transparency, it is necessary to attach magnetic devices to microcavities to ensure that the whole sensing units are subjected to magnetic force in the magnetic fields. When applying an AC signal with a high frequency of *f*_1_ to a coil with certain number of turns, according to Ampere’s law, the signal excites an AC magnetic field, which can be regarded as an electromagnet with periodically varying magnetic poles. When the energized coil is placed in an externally applied AC magnetic field with a low frequency of *f*_2_, it will be subjected to a magnetic field force that constantly changes direction and magnitude due to the signal frequency.

To measure low-frequency magnetic fields, as shown in Figure 1b, a composite structure with an excitation coil and an optical microcavity is designed. Essentially, when measuring low-frequency magnetic field signals, the coil attached to the microcavity serves as the receiving coil, generating a magnetic signal with a frequency of *f*_1_, which can be understood as a carrier signal within the framework of modulation and demodulation techniques. The external magnetic signal with a low frequency of *f*_2_ is regarded as a modulating signal. The interaction between the two magnetic fields propagates force to the sensing unit, transforming into a constantly changing squeezing or stretching elastic force applied by the receiving coil to the microcavity, causing the mechanical vibration frequency of the microcavity to vary according to the following equations.

(1) $$S_{1}=A_{1}\times \sin(2\pi f_{1}t)$$

(2) $$S_{2}=A_{2}\times \sin(2\pi f_{2}t)$$

(3) $$S=S_{1}\times S_{2}=\frac{A_{1}A_{2}}{2}\times \{\cos[2\pi (f_{1}-f_{2})t]-\cos[2\pi (f_{1}+f_{2})t]\}$$

*S*_1_ and *S*_2_ respectively represent the carrier magnetic signal generated by the receiving coil and the modulating magnetic signal to be measured; *A*_1_ and *A*_2_ are the amplitudes of the two magnetic signals, respectively; and *f*_1_ and *f*_2_ are the frequencies of the magnetic signals, respectively, and conform to *f*_1_ >> 100 *f*_2_. The microcavity will vibrate at the AM signal frequency *f*_1_ ± *f*_2_ (Figure 1c). Vibration within the constraints of the copper column induces the mechanical deformation of the microcavity, leading to changes in its radius and effective refractive index, thereby altering the resonant mode. Variations in the radius, effective refractive index, and resonant mode wavelength satisfy the WGM standing wave condition equation:

(4) $$2\pi Rn_{eff}=m\lambda_{r}$$

where *R* is the radius of the microcavity and *n_eff_* is the effective refractive index, both of which change due to the force acting on the microcavity. *m* is the number of resonant modes, and *λ_r_* is the resonant mode wavelength of the laser.

Our previous research confirmed that the change in the microcavity radius under the action of mechanical force is the main influencing factor, while the effect of the effective refractive index can be ignored [10]. Mechanical vibration causes periodic changes in resonant modes, and weak, low-frequency magnetic signals are modulated to preserve their frequency and phase characteristics in the information contained in the transmission spectrum. After laser frequency locking, the periodic frequency shift of the resonant wavelength is reflected in the variation in the transmitted optical field intensity in the cavity. Through the lock-in amplification technique, the transmission spectrum is demodulated with the carrier signal frequency and filtered to separate and restore the signal with the same frequency as the modulating signal but of a larger amplitude. This method of detecting weak low-frequency signals through modulation and demodulation extends the application range of magnetic sensing in microcavities, extending the detectable frequency range to hertz and millihertz, no longer being limited to the eigenfrequency of the microcavity.

**Figure 2:**
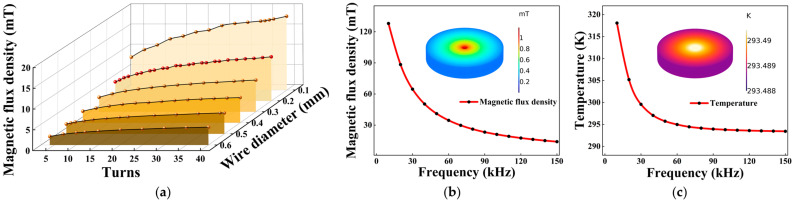
(**a**) Influence of different turns and wire diameters on the magnetic flux density of the coil. (**b**) Variation in coil magnetic flux density for different signal frequencies. (**c**) Variation in coil temperature for different signal frequencies.

The selection of a suitable receiving coil is also noteworthy. We first need to determine the structure of the coil and how to constrain the thermal and magnetic properties it exhibits under high-frequency excitation. First, we establish a coil domain model in finite element simulation analysis software COMSOL Multiphysics 6.0 and set the wire section to circular. A spherical shell representing the air domain is added outside the coil, and the boundary condition is set as “infinite element domain”. The coil is divided by the refined triangular sweep mesh, and the rest is the default free tetrahedral mesh. By setting physical fields such as “magnetic fields”, “heat transfer in solids”, and multiphysics coupling such as “electromagnetic heating”, the software will automatically call the relevant equations for simulation. Then, we separately analyze the turns and wire diameter of the receiving coil (Figure 2a). When the same excitation is applied, the coil models corresponding to each wire diameter increase from 5 turns to 40 turns, and the magnetic flux density at the center of the receiving coil is obviously enhanced with the increase in the number of turns. When the number of turns is the same, a finer wire diameter reduces the overall size of the coil and increases the magnetic flux density, in line with the formula *B* = *μ*_0_*I*/2*r*, where *μ*_0_ is the vacuum magnetic permeability, *I* is the current in the coil, and *r* is the coil radius.

We add the “coil geometry analysis” and “frequency domain steady state” conditions, as well as a parametric sweep of the excitation signal frequency, and solve the model. Under high-frequency excitation, the central magnetic flux density of the coil shows a decreasing trend with increasing frequency (Figure 2b), which originates from the decrease in the internal current of the coil. According to Joule’s law, the temperature of the coil also changes correspondingly, and the simulation results also prove this (Figure 2c). With a preset temperature of 293.15 K and an applied voltage of 5 V, under ideal heat flux conditions, the steady-state temperature decreases by 19 K when the frequency increases from only 10 kHz to 30 kHz. At around 150 kHz, the steady-state temperature is only about 0.5 K higher than the preset temperature. The CaF_2_ microcavity is extremely sensitive to temperature variations, while the high-frequency current reduces the influence caused by thermal effects. The simulation work verifies the feasibility of the scheme, taking into account both the turns and wire diameter when selecting coils. When excited by high-frequency signals, the coil becomes the ideal carrier for the AC magnetic field.

## 3. Fabrication

**Figure 3:**
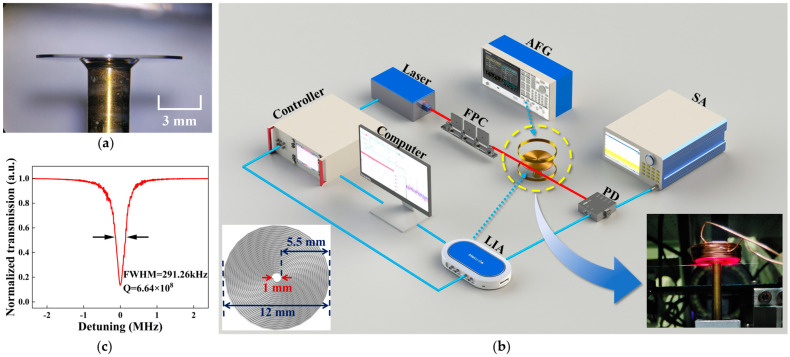
(**a**) Side view of the microcavity. (**b**) Schematic diagram of the magnetic sensing experimental system; the red line indicates the optical path, and the blue line indicates the circuit. AFG: arbitrary function generator; SA: spectrum analyzer; FPC: fiber polarization controller; PD: photodetector; LIA: lock-in amplifier. Inset 1 shows the geometric definition of the receiving coil. Inset 2 represents a multilayer-structure sensing unit and the use of visible light to simulate coupling with the microcavity. (**c**) Lorentz line shape of the normalized transmission spectrum.

We select a CaF_2_ crystal with a diameter of 10 mm and a thickness of 0.2 mm for polishing to prepare the microcavity and then tightly fix it to the air-bearing spindle fixture with extremely low radial and axial vibrations through a copper column. We then polish the cylindrical surface of the microcavity with a diamond abrasive solution until its surface roughness is reduced to the sub-nanometer level (Figure 3a). Polishing reduces the light propagation loss on the cylindrical surface of the CaF_2_ crystalline microcavity, making it easy to couple with the waveguide and form the WGM.

The experimental system is shown in Figure 3b. A certain wavelength of laser energy is output from a tunable laser, which is coupled with the microcavity by a tapered fiber through an evanescent field after the polarization state and optical power are adjusted by a fiber polarization controller (FPC). At the end of the fiber, the optical signal is converted into an electrical signal through a photodetector (PD) and output to a spectrum analyzer (SA) and a lock-in amplifier (LIA). The former is used to observe the PSD signal in the frequency domain, and the latter is used to provide the carrier signal and demodulate the output voltage amplitude signal. Finally, through the feedback loop, the signal is applied to the laser controller, and the computer is used to execute signal processing. The varying laser frequency hinders our analysis of the specific frequency response. In order to realize the observation of PSD and match the response corresponding to different eigenfrequencies, the Pound–Drever–Hall (PDH) technique needs to be used for frequency locking. The PDH technique dynamically changes the laser frequency to match the specific frequency of the microcavity WGM by adjusting the proportional, integral, and differential parameters, stabilizing the frequency of the tunable laser at the corresponding value of the mode with the highest Q factor.

In order to facilitate the clamping and power supply to the receiving coil’s wire, combined with the simulation results, we choose a coil wire diameter of 0.2 mm, an outer ring diameter of 12 mm, and an inner diameter of 1 mm. A single layer contains about 27 turns, with a total of 15 layers, and each layer is connected in series. The flexibility of the coil’s ability to apply signals extends the sensor’s ability to be used in different external magnetic field conditions. The coil and the microcavity are bonded by UV glue to form a sensitive unit. The sensing unit and a tapered optical fiber with a diameter about 1 μm are fixed on two manual stages. We first couple a red light to the microcavity for indicating, as shown in the inset of Figure 3b, and determine the coupling position. A laser with a center wavelength of 1550 nm is used to couple with the microcavity to excite WGM resonance and obtain a Lorentz line shape, as shown in Figure 3c, and the Q factor of the microcavity is calculated by the full width at half-maximum (FWHM) method. The results of the experiment show that the FWHM is 291.26 kHz, and the corresponding Q factor is 6.64 × 10^8^. Although the Q factor usually decreases due to environmental factors during coil assembly, it generally maintains a high level.

The AC signal is applied through the coil’s lead wire, which needs to be kept slack during the test in order to avoid excessive stress affecting the transmission of the magnetic field force. The sensing unit is placed parallel at the center of a Helmholtz emitting coil with a known coil constant, and the emitting coil generates a uniformly distributed magnetic field with a magnetic induction line perpendicular to the sensitive unit, such that an accurate external magnetic flux density can be obtained by measuring its current. The receiving and emitting coils are connected to the LIA and a high-precision arbitrary function generator (AFG), respectively, and by changing the signal of the emitting coil, the testing of magnetic field signals with different frequencies can be accomplished in the same system. In order to reduce the interference of environmental factors such as geomagnetic field and electromagnetic wave radiation in instruments, the experiment will be carried out in a magnetic shielding device. The test system can be improved in combination with non-Foster electronics or a transient parity–time symmetry system to improve the high-frequency performance of the receiving coil or the anti-interference ability of the system [27,28].

## 4. Experiment

**Figure 4:**
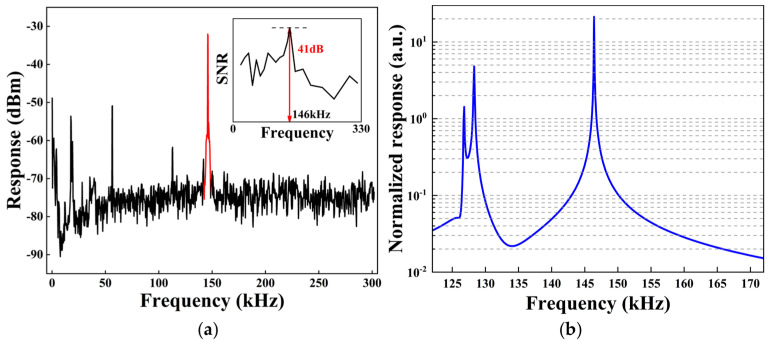
(**a**) PSD shown by the spectrum analyzer when loaded with a 146 kHz signal; the inset represents the variation in SNR with frequency. (**b**) Simulation result of the microcavity’s mechanical mode frequency response.

In order to obtain a stronger response and higher modulation efficiency, we need to determine the overall mechanical resonant frequency distribution of the sensitive unit. First, the Helmholtz emitting coil is applied with a direct current signal to generate a constant static external magnetic field, and a sinusoidal AC signal with frequency scanning in the range of 20 kHz to 300 kHz is applied to the receiving coil. Under the action of the magnetic field force, the microcavity will directly generate mechanical resonance at AC frequency, and the change in the transmission spectrum will also be reflected in the SA and LIA. The PSD of the signal after PDH frequency locking generates the main peak of the response at the corresponding frequency (Figure 4a), and the difference between the peak and the noise floor (i.e., signal-to-noise ratio, SNR) reflects the responsiveness of the system to the magnetic field. The noise suppression effect of our scheme is mainly reflected through the use of LIA.

From the inset in Figure 4a, it can be seen that the mechanical resonance response is strongest, at about 41 dB, when the AC frequency is 146 kHz. The characteristic frequency of the sensing unit composed of a coil and a microcavity is simulated and analyzed, and the strongest frequency response is also achieved at 146 kHz (Figure 4b). Compared with other resonant frequencies, 146 kHz gives the microcavity the highest frequency response amplitude. In order to maximize the force and deformation of the microcavity and to obtain the highest possible sensitivity, a 146 kHz and 10 V peak-to-peak AC signal is applied to the receiving coil in the experiment according to the frequency scanning and simulation results. After the signal is applied for a period of time, the magnetic flux density and temperature characteristics of the receiving coil are verified by using a high-precision magnetometer and thermometer, and the results are similar to the simulation results.

**Figure 5:**
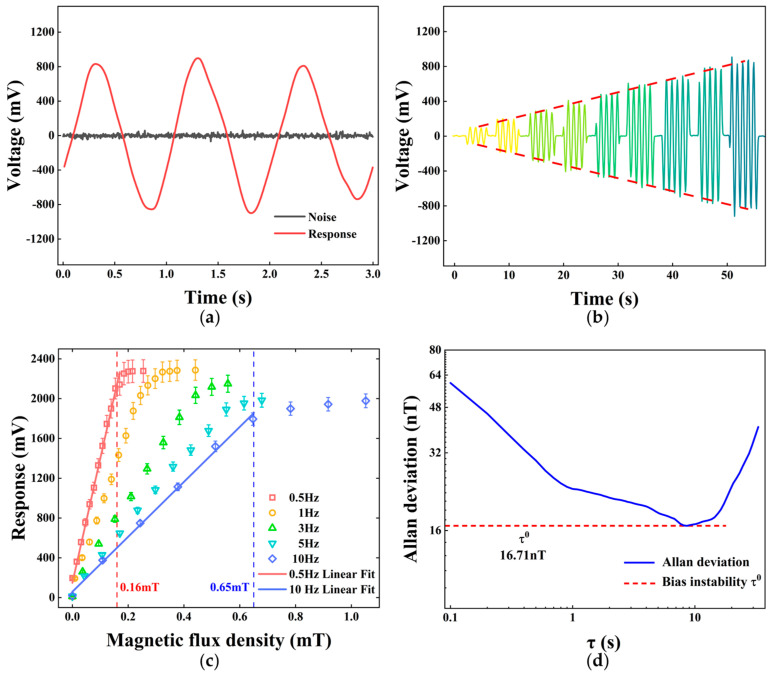
(**a**) System response to zero-input noise and a 1 Hz signal. (**b**) Response step signal of the AC magnetic fields with gradually increasing amplitude over time. (**c**) System response output as a function of the magnetic signal with different frequencies. (**d**) Allan deviation fitting of the system.

Next, we verify the ability of the sensor to test the AC magnetic field signals. As shown in Figure 5a, when the emitting coil is not loaded with a signal, the demodulated output of the system is close to a straight line. The amplitude of the straight line at this time corresponds to the zero-input noise floor of the sensing system, which we reduce to about 9.14 mV using the filtering function of the LIA. When the AFG loads the emitting coil with a 1 Hz AC signal, a clear sinusoidal response of the same frequency can be seen, and the peak-to-peak value is taken as the demodulated output signal amplitude of the system. Figure 5b shows the response step signal obtained by gradually increasing the amplitude of the 1 Hz signal over time. After filtering, the low-frequency noise is not obvious, which better restores the frequency information of the magnetic test signal, and the change in peak-to-peak value exhibits a certain linear trend.

We subsequently measure low-frequency magnetic signals ranging from 0.5 Hz to 10 Hz, gradually increasing the AFG output amplitude until the output signal generates saturation and cutoff values due to the performance limitation of the LIA. The output signal will show nonlinearity due to the cutoff of the amplitude growth process, which is also caused by a rapid reduction in the transmission spectrum slope of the microcavity with frequency detuning [29]. The external magnetic flux density is calculated by synchronously measuring the current value of the emitting coil using a high-precision digital multimeter. In order to ensure the accuracy of the test results and the repeatability of the experiments, we conduct multiple experiments, fit the linear portion of each frequency measurement point to a straight line (Figure 5c), and use its slope to represent the sensitivity. After three repeated experiments, the measurement results show a gradual decrease in the sensitivity of the system as the signal frequency is increased, with a sensitivity of 12.49 V/mT for the 0.5 Hz magnetic field and 2.76 V/mT for the 10 Hz magnetic field. This phenomenon stems from the fact that the lower modulation frequency makes the frequency difference between the AM signal and the carrier signal smaller, which is still within the bandwidth, where the main peak response decreases by 3 dB. Moreover, the lower the modulation frequency, the higher the original intensity of the AM signal. In the experimental process, a higher Q factor corresponds to a steeper Lorentz line shape, and a more pronounced change in the optical field intensity can be obtained at the same frequency shift, so increasing the Q factor can significantly improve the sensitivity of the magnetometer. In addition, the LC resonant circuit composed of a specific capacitor connected in series with the receiving coil can offset the impact caused by most of the coil inductive reactance, and only a very low excitation signal voltage is required to achieve the same level of sensitivity, which is also the main goal for our future optimization.

The bias stability of the system without the test magnetic field is also an important index to measure the performance of the sensor. Due to the joint action of the magnetic shielding device and the signal filtering and noise reduction function, the sensor should have good bias stability. We use the Allan deviation fitting double logarithmic diagram for verification (Figure 5d), and the results show that the bias instability noise, i.e., the corresponding ordinate of the point with a slope of zero, in the diagram is 16.71 nT, indicating that the system has good low-frequency magnetic sensing performance.

## 5. Conclusions

In this experiment, we design and fabricate a WGM crystalline microcavity magnetometer for low-frequency AC magnetic sensing. The principle of cavity resonant mode change caused by magnetic signal modulation between two sets of coils is analyzed theoretically, and the coil structure is determined by a finite element simulation analysis of magnetic flux density and temperature. The periodic deformation and frequency shift of the microcavity are realized through the interaction of field force with the AC magnetic field. We demonstrate a sensitivity of 12.49 V/mT for a 0.5 Hz AC magnetic field by frequency locking and lock-in amplification techniques. The bias instability noise of the system is as low as 16.71 nT. The sensitivity of the magnetometer is mainly affected by the optical Q factor and the magnetic flux density generated by the receiving coil. The properties of the magnetometer can be improved by preparing a microcavity with a smoother cylindrical surface, increasing the coil excitation voltage, or constructing an LC resonant circuit. Our magnetometer expands the application of microcavities for high-sensitivity testing of low-frequency AC magnetic fields. After optimization, the measurable frequency is expected to be further reduced. The magnetometer proposed in this work has two possible development trends in the future. First, by using a receiving coil that can generate a stronger magnetic flux density we can sacrifice the small size advantage of the sensing unit and improve the amount of frequency shift and response strength by increasing the force and deformation. Second, we can plate the metal wire on the surface of the microcavity through micro–nano manufacturing technology to form a planar coil. This method improves the integration of the sensing unit and provides the possibility for small-volume packaging and on-chip integration but greatly weakens the magnetic flux density of the receiving coil and poses new challenges to the method of applying electrical signals.

## Data Availability

The original contributions presented in this study are included in the article. Further inquiries can be directed to the corresponding author.
